# Reliability, validity, and minimal clinically important differences for the Thai-version of the Aberdeen Varicose Vein Questionnaire (AVVQ-Thai) in patients with chronic venous disease

**DOI:** 10.12688/f1000research.147716.2

**Published:** 2024-10-11

**Authors:** Boonying Siribumrungwong, Pinit Noorit, Termpong Reanpang, Chaowanun Pornwaragorn, Chumpon Wilasrusmee, Suchat Wongsuwanich, Kanoklada Srikuea, Saritphat Orrapin, Thoetphum Benyakorn, Andrew Malcolm Garratt, Kittipan Rerkaserm

**Affiliations:** 1 Center of Excellence in Applied Epidemiology, Faculty of Medicine, Thammasat University, Pathum Thani, 10120, Thailand; 2 Division of Vascular Surgery, Department of Surgery, Faculty of Medicine, Thammasat University, Pathum Thani, 10120, Thailand; 3 Department of Surgery, Chonburi Hospital, Mueang Chonburi District, Chon Buri, Thailand; 4 Department of Surgery, Faculty of Medicine, Chiang Mai University, Chiang Mai, Chiang Mai, Thailand; 5 Department of Surgery, Faculty of Medicine, Ramathibodi hospital, Mahidol University, Bangkok, Thailand; 6 Language Institute, Thammasat University, Pathum Thani, Thailand; 7 Division for Health Services, Norwegian Institute of Public Health, Oslo, Norway; 8 Clinical Surgical Research Center, Department of Surgery, Faculty of Medicine, Chiang Mai University, Chiang Mai, Thailand; 9 Environmental - Occupational Health Sciences and Non-Communicable Diseases Research Center, Research Institute for Health Sciences, Chiang Mai University, Chiang Mai, Thailand

**Keywords:** AVVQ, Aberdeen Varicose Vein Questionnaire, reliability, validity, minimal clinically important differences, venous, reflux

## Abstract

**Background:**

Patient-reported outcome measures (PROMs) are essential for assessing the health of patients with chronic venous disease (CVD). Therefore, we aimed to translate the Aberdeen Varicose Vein Questionnaire into Thai language (AVVQ-Thai) and evaluate its reliability and validity. Minimal clinically important differences (MCID) of the AVVQ-Thai also be estimated.

**Methods:**

International standards for PROM translation were followed including the forward-backwards translation of the AVVQ. Patients with Clinical-Etiology-Anatomy-Pathophysiology (CEAP) C2-C6 with truncal reflux were prospectively included. Venous interventions were used to treat reflux and varicosities. Patients’ characteristics, venous clinical severity scores (VCSS), EuroQol EQ-5D, and AVVQ-Thai were collected pre- and one-month post-intervention. AVVQ-Thai was also collected one to two weeks after the initial visit by reply-paid postal questionnaire.

**Results:**

The study included 119 patients (30% C2, 29% C3, 28% C4, 11% C5, and 2% C6). The AVVQ-Thai had good internal consistency with Cronbach’s alpha of 0.783 and moderate reliability with the intraclass correlation coefficient of 0.67 (95%CI: 0.50, 0.79). The AVVQ-Thai was significantly correlated with VCSS and was able to discriminate patients with different levels of health problems as assessed by EQ-5D at both pre-and post-intervention, demonstrating good construct and discriminative validity. The median AVVQ scores improved significantly after intervention from 15.4 (IQR 8.3, 24.2) to 4.2 (IQR 1.3, 8.4) in C2-C3, and 18.9 (IQR 14.1, 25.5) to 7.3 (IQR 4.6, 16.3) in C4-C6. The MCID of the AVVQ was 6.21 on the 0-100 scale, which equates to the level of difference necessary to be clinically meaningful.

**Conclusions:**

AVVQ-Thai has satisfactory evidence for internal consistency, reliability, validity, and responsiveness to change and is recommended for application in Thailand.

## Introduction

Chronic venous disease (CVD) is common in the general population, with the prevalence varying from 17-40%, ^1^ and affects the quality of life of patients. ^2^^,^^3^ Management includes medication, compressive stockings, ablation of superficial and perforator vein reflux, and treatment of iliocaval venous obstruction. ^4^^,^^5^ Outcomes of treatment include hemodynamic success (i.e., occlusion of the ablated refluxed vein), venous clinical severity score (VCSS), and self-reported health and quality of life (QoL) using patient-reported outcome measures (PROMs). ^4^

PROMs are important for patient-centered approach and useful for helping detect change after treatment and comparing treatment effects between different interventions. Several PROMs have been evaluated and recommended as assessment tools in patients with CVD, including generic (e.g., SF-36, EuroQol EQ-5D) and venous-specific QoL measurements (e.g., Aberdeen Varicose Vein Questionnaire (AVVQ), Chronic Venous Insufficiency questionnaire (CIVIQ), and Venous Insufficiency Epidemiological and Economic study (VEINS)). ^4^^,^^6^

Generic PROMs such as the EQ-5D can contribute to cost per quality adjusted life year calculation as part of economic evaluation. Venous-specific PROMs are of clinical relevance and sensitive to small but important changes in health and differences between patients. ^3^ Our earlier study in Thai patients with CVD found significant improvement of generic QoL after venous intervention, measured by EQ-5D. ^7^ However, 12% of the patients already had the highest possible EQ-5D scores at pre-intervention, which limits responsiveness to change. ^7^

AVVQ is the most rigorously evaluated PROM in patients with varicose veins. ^8^ It demonstrated good validity, reliability, and responsiveness and had similar performance across countries. ^9^^–^^11^ Validity is the degree to which the tool measures the construct it purports to measure, whereas reliability is consistent of the measures, while responsiveness is the ability of the tool to detect change over time. ^12^ Nevertheless, differences in language, culture, and values between Thailand and European countries should be considered before application of the AVVQ in Thai patients. ^13^ Furthermore, minimal clinically important difference (MCID), defined as the smallest change perceived as beneficial or meaningful by the patient, is important for interpretation of the scores in clinical settings. However, to date, there is still insufficient study in this area. ^14^

Currently, there is no validated translated venous-specific questionnaire in Thailand. Therefore, this study aimed to translate the AVVQ into the Thai language and assess the reliability and validity of the Thai-translated version (AVVQ-Thai). In addition, we also estimated minimal clinically important differences (MCID) for the AVVQ score.

## Methods

A multicenter prospective observational cohort study was conducted from January 2019 to Dec 2021 in 4 hospitals across Thailand, which included Thammasat University Hospital in the Central, Maharaj Nakorn Chaing Mai Hospital in the North, Ramathibodi in the Central, and Chonburi Hospital in the East of Thailand. The study was approved by all study center’s ethic committees (The Human Research Ethics Committee of Thammasat University (Medicine) with approval number MTU-EC-SU-1-130/61 (approved date July 26, 2018) for Thammasat University Hospital; The Research Ethics Committee 4 of Faculty of Medicine, Chiang Mai University with approval number SUR-2562-06329 (approved date June 28, 2019) for Maharaj Nakorn Chaing Mai Hospital; The Office of The Committee for Research, Faculty of Medicine Ramathibodi Hospital Mahidol University with approval number MURA2019/173 (approved date March 13, 2019 for Ramathibodi Hospital; The Research Ethic Committee of Chonburi Hospital with approval number 26/62/S/h3 (approved date March 20, 2019) for Chonburi Hospital. The study was adhered to the Declaration of Helsinki.

The study conformed with the COSMIN reporting guideline for studies on measurement properties of patient-reported outcome measures. ^15^ The reporting checklist was uploaded as extended data in the online database. ^16^

### Participants

The study included adults of ≥18 years of age able to read Thai and Clinical-Etiology-Anatomy-Pathophysiology (CEAP) C2 to C6 with symptoms related to the venous disease and unilateral pathologic reflux of great (GSV) or small saphenous veins (SSV) who would undergo venous intervention. Exclusion criteria were concomitant peripheral arterial disease, morbid obesity (body mass index ≥ 35 kg/m ^2^), pregnancy, recurrence varicose vein, history of previous venous intervention, and history of deep vein thrombosis or presence of deep vein obstruction from duplex scan. All participants signed informed consent before entering the study.

### Aberdeen Varicose Vein Questionnaire (AVVQ)

AVVQ is a clinically derived questionnaire specifically developed for patients with varicose veins. First developed and tested for measurement properties by Garratt et al. in 1993, ^3^ it comprises four domains (pain and dysfunction, cosmetic appearance, extent of varicosity, and complications) with 13 questions that are summed based on a clinical weighting and scored from 0 to 100, where higher score represent greater CVD severity. ^3^^,^^11^

### Translation process ( Figure 1)

**Figure 1.**
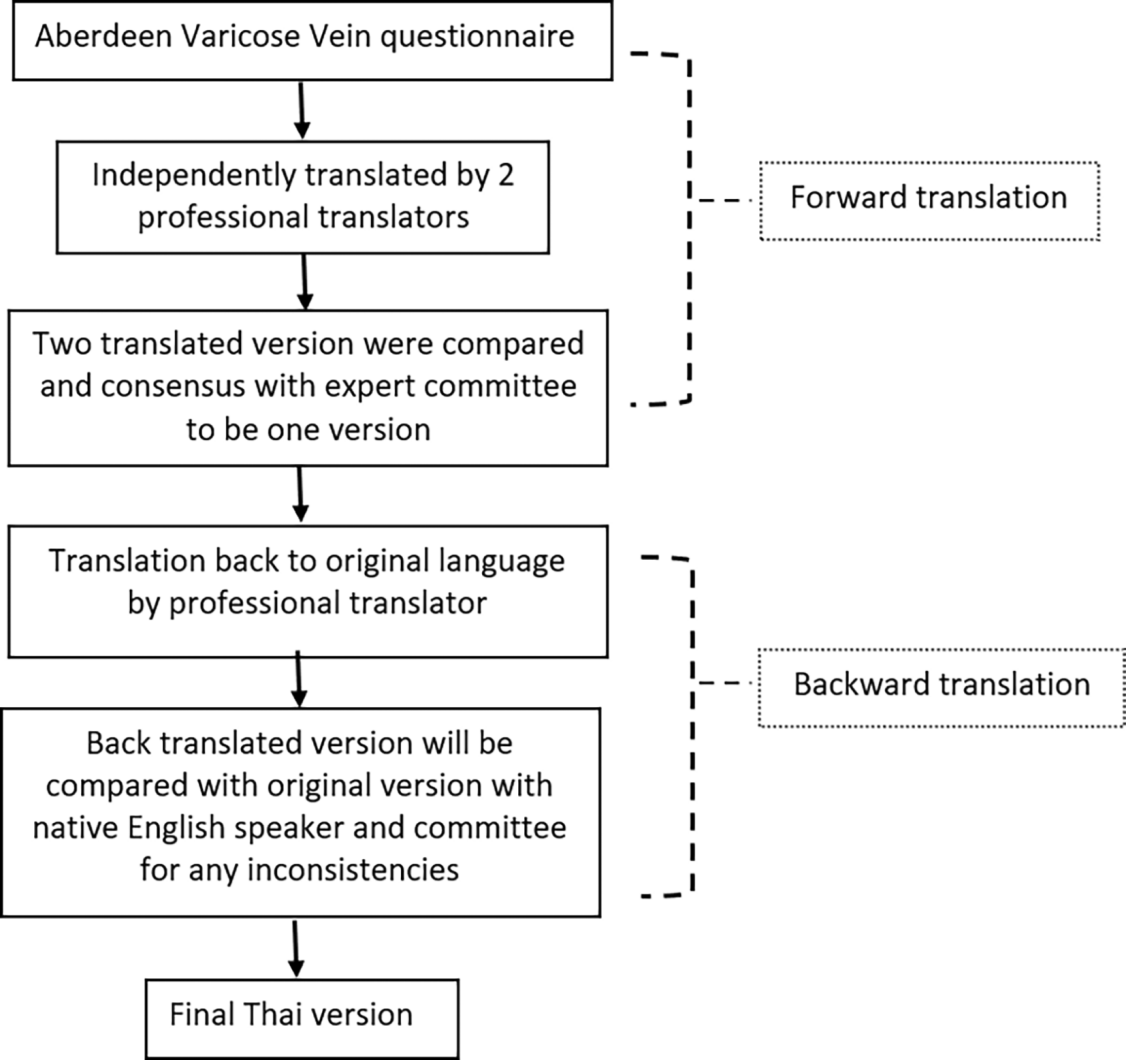
Forward and backward translation process of the AVVQ.

SW and a team form the Language Institute of Thammasat University undertook translation which followed recommendations by the European Organization for Research and Treatment of Cancer. ^17^ First, the English version of AVVQ was independently translated into Thai by two professional Thai translators, trying to make the language clear and as simple as possible. Then, the research team assessed both translations for equivalence with the original one. Any discrepancies were resolved by consensus and resulted in a single provisional forward translation. After that, the provisional Thai forward translation was back translated to English by another native English speaker and compared with the original English version to ensure equivalence. The final translated Thai version was reviewed by the research team and was used in the study. The English translated and final translated Thai versions were uploaded online as extended data and were available at https://doi.org/10.5281/zenodo.10990113. ^16^

### Main survey and outcome measurement

Baseline characteristics, EQ-5D-3L, preoperative AVVQ-Thai, and revised venous clinical severity scores (VCSS), ^18^ were obtained at an initial outpatient visit using a self-completed pen and paper questionnaire and clinician completion for VCSS. For purposes of assessing test-retest reliability, patients took a second AVVQ-Thai questionnaire and reply-paid envelope home for completion 1 to 2 weeks later (retest AVVQ-Thai). After the venous intervention, postoperative complications, EQ-5D-3L, postoperative AVVQ-Thai, and VCSS were collected one-month post-intervention. Mean scores of the preoperative and retest AVVQ-Thai were used as preoperative scores in the analysis. In the case of missing data for individual AVVQ questions, data from the preoperative or retest questionnaires were substituted accordingly.

### Interventions

Refluxed veins were ablated using either endovenous thermal ablation (i.e., radiofrequency (RFA), endovenous laser ablation (EVLA)), or surgical therapy (saphenofemoral ligation with stripping for GSV or SPJ ligation for SSV) depending on patients’ preferences and costs incurred. All procedures were performed under regional anesthesia. Briefly, radiofrequency ablation was performed with tumescent anesthesia to ablate the GSV at 2–3 cm distal to the saphenofemoral junction to the GSV around the knee level. In surgical therapy, saphenofemoral junction was ligated flush to the common femoral vein. Then, GSV was stripped from proximal to distal GSV around the knee level. Pathologic perforator insufficiency was treated by ultrasound-guided foam sclerotherapy in CEAP C5-6. Varicosities would be treated concomitantly by either phlebectomy or foam sclerotherapy. ^4^

### Statistical analysis

Sample size is estimated using the rule of thumb for questionnaires testing. At least 10 subjects were necessary for 1 question with a minimum number of 100 subjects to ensure the stability of the variance-covariance matrix. ^19^ As a result, with 13 questions in AVVQ so 130 patients were necessary. Accounting for 10% loss follow up rate, 145 patients would be recruited.

Cronbach’s alpha and intraclass correlation coefficient (ICC) were used to assessed internal consistency and test-retest reliability of the AVVQ-Thai, respectively. Cronbach’s alpha reflects the homogeneity of the AVVQ questions in assessing the same concepts. Higher values reflect higher levels of consistency/homogeneity with level of 0.70 and above being satisfactory. ^20^ ICC and 95%CI were estimated by a two-way mixed effect model with an absolute agreement for the overall scores and within each item of the AVVQ. Values of less than 0.5, 0.5 – 0.75, 0.75 – 0.9, and greater than 0.9 indicate poor, moderate, good, and excellent reliability, respectively. ^21^

Hypothesis testing was used to assess the construct validity of the AVVQ-Thai including correlations of the AVVQ scores and VCSS both pre- and postintervention, and comparing median AVVQ scores between patients with different degrees of problems reported each dimension of EQ-5D (no problem vs. some and severe problems). It was hypothesized that AVVQ scores would have low to moderate correlations with the VCSS because while there is some content overlap, the latter is completed by the clinician and is more concerned with symptoms than how varicose veins affect quality of life. It was hypothesized that patients with higher scores on the AVVQ would report more problems across the five EQ-5D dimensions. Responsiveness of the AVVQ-Thai was assessed by determining the change in the AVVQ scores after intervention according to the CEAP clinical class (C2-C3 and C4-C6). Ceiling effects, or the proportion of patients with the best possible scores that cannot be improved with intervention, were also compared for the AVVQ and EQ-5D.

Distribution-based methods were used to estimate minimal clinically important differences (MCID) of the AVVQ. ^22^^,^^23^ There are several methods of MCID derivation. Four types of methods were analyzed and reported for consideration. 1) 0.5 of SD of the observed change (SD _observed change_) in AVVQ as recommended following a systematic review by Norman et al. ^24^ 2) Standard error of measurement (SEM), which represents the variability of the scores. Any score difference below the SEM is likely due to measurement error rather than genuine change. Thus, it is the most conservative value of MCID. SEM was calculated by the equation $\mathrm{SEM}=\mathrm{SD}\;\text{of baseline score}\times \sqrt{(1}-r)\;$ where *r* = the intraclass correlation coefficient. 3) Minimum detectable change (MDC) is calculated from SEM. MDC _95_ is the measurement that has 95% confidence that this value is more than the measurement error. The equation is MDC=SEM×1.96×√2 . 4) MCID derived from effect size (ES) by multiplying SD of the baseline scores with 0.2, which is the accepted value for small ES.

Analysis was undertaken in STATA version 16.1 and a p value of less than 0.5 considered as statistically significant. Missing data was excluded from the analysis. Spearman’s correlation was used to determine correlation. The Wilcoxan rank-sum test, and the Wilcoxan matched-pairs signed-rank test was used to compare median scores for independent and dependent continuous data, respectively.

## Results

A total of 119 patients were recruited, including 67 (56%) from Thammasat University, 25 (21%) from Chonburi, 19 (16%) from Maharaj Nakorn Chaing Mai, and 8 (7%) from Ramathibodi hospital. Seventy-six (64%) patients were female, with a mean age of 56 years (SD = 13) and a BMI of 26 kg/m ^2^ (SD 5). Most patients were in Clinical-Etiology-Anatomy-Pathophysiology (CEAP) Clinical class 2 (30%), followed by C3 (29%), C4 (28%), C5 (11%), and C6 (2%). Most patients had GSV reflux (94%), while the remainder had short saphenous vein reflux. There were 43 (36%) patients who had completed a university education.

**Figure 2.**
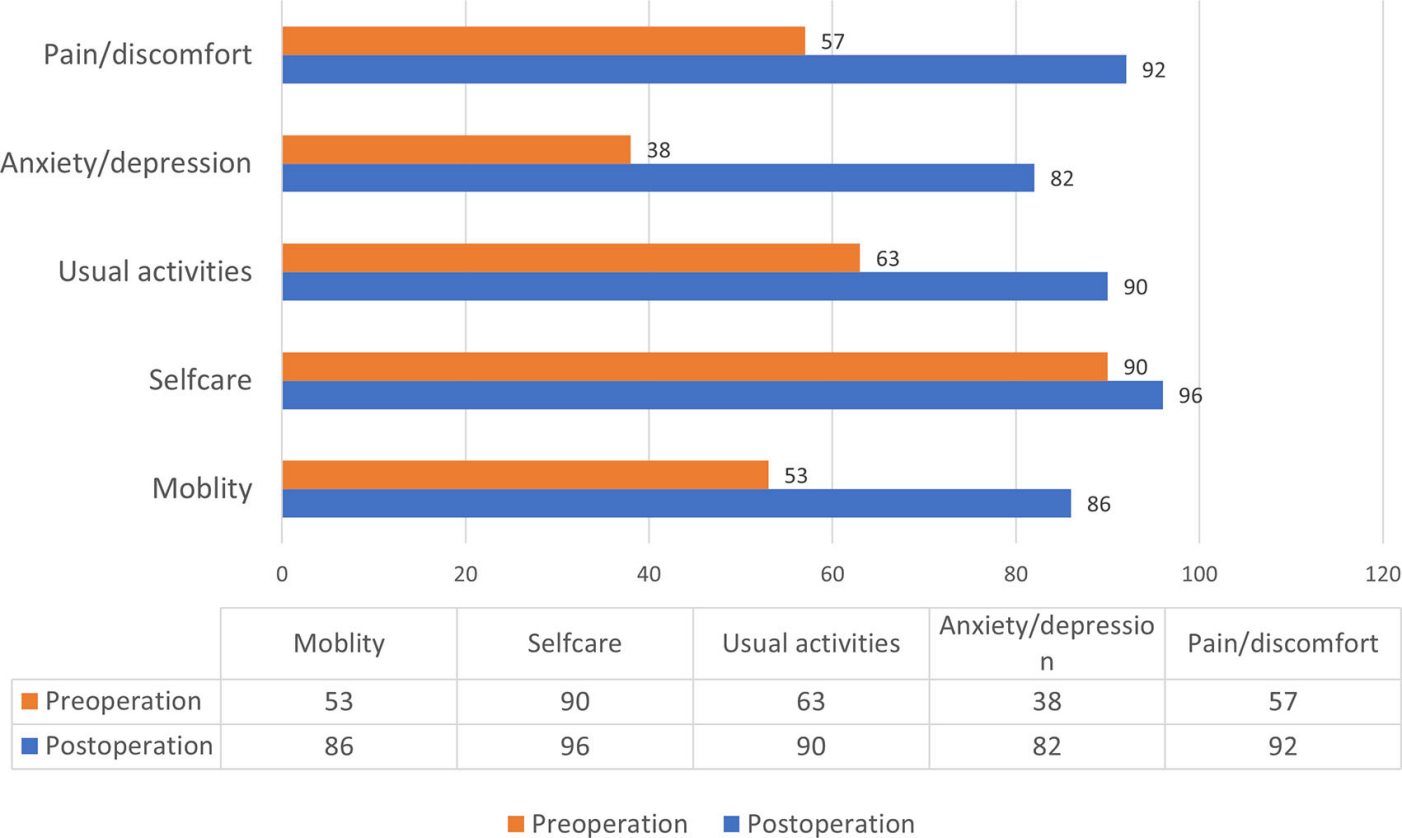
Proportion of patients reporting no problem by dimension of EQ-5D.

All patients had successful operations (i.e., uneventful operations), including 47 (40%) RFA, 11 (9%) EVLA, and 61 (51%) open surgery. The mean VCSS scores improved from 6.7 (SD = 3.2) preoperatively to 2.7 (SD 2.3) post-operation, with a mean difference of 4 (95%CI: 3.6, 4.5). ( *p* < 0.001). One hundred eighteen (99%) and 116 (97%) patients completed preoperative and postoperative EQ-5D, respectively. The proportion of patients reporting no problems was significantly less in across all but the self-care dimension ( Figure 2).

### AVVQ

The translation process resulted in the Thai-version of the AVVQ (AVVQ-Thai). ^16^ One hundred and ten (92%), 94 (79%), and 112 (94%) patients completed the preoperative, retest, and postoperative AVVQ-Thai, respectively. The mean AVVQ scores improved from 18.9 (standard deviation (SD) = 10.9; interquartile range (IQR) 10.1, 24.9) preoperatively to 7.8 (SD = 7.6; IQR 2, 11) at 1-month post operation ( *p* < 0.001) with the mean difference of 11.1 (95%CI: 8.8, 13.4).

***Reliability***

Cronbach’s alpha of the preoperative and postoperative AVVQ were 0.783 and 0.790, demonstrating acceptable internal consistency. The overall ICC for the total scores was 0.67 (95%CI: 0.50, 0.79), demonstrating moderate reliability. Item No.8 had the lowest ICC of 0.51 (95%CI: 0.27, 0.67), while item No.3 had the highest ICC of 0.85 (95%CI: 0.79, 0.90). All items had ICCs that were more than 0.5, and 3 out of 13 (23%) had ICCs that were more than 0.75 ( Table 1).

**Table 1.** Intraclass correlation coefficients of the test-retest reliability according to each item of the AVVQ.

Item	Descriptions	ICC	95%CI
1	Draw your varicose veins on the follow images	0.58	0.38-0.72
2	How many days in the past 2 weeks have you had pain or aching of your varicose veins?	0.70	0.55 – 0.80
3	How many days in the past 2 weeks have you taken painkillers medicines for your varicose veins?	0.85	0.79 – 0.90
4	How much leg swelling have you had in the past two weeks?	0.78	0.68 – 0.85
5	Have you worn stockings or such in the past 2 weeks?	0.83	0.75 – 0.89
6	Have you had any itching in the past 2 weeks because of your varicose veins?	0.65	0.48 – 0.77
7	Have you had a violet colored skin changes due to small vessel caused by your varicose veins?	0.59	0.39 – 0.72
8	If you have any redness or inflammation around the ankle?	0.51	0.27 – 0.67
9	Have you been sad in connection with your varicose veins?	0.57	0.36 – 0.71
10	Do you get worried because of your varicose appearance?	0.72	0.58 – 0.81
11	Affected your dress (including the use of pantyhose) by dark varicose veins?	0.61	0.42 – 0.73
12	Has your house work or other daily activities been influenced by your varicose past 2 weeks?	0.70	0.55 – 0.79
13	Have your leisure activities (including sports, hobbies, and social life) become influenced by your varicose past 2 weeks?	0.63	0.46 – 0.75
	Overall	0.67	0.50 – 0.79

AVVQ, Aberdeen Varicose Vein Questionnaires; CI, confidence interval; ICC, intraclass correlation coefficient.

***Validity***

Both pre-and postoperative AVVQ were significantly correlated with pre- and postoperative VCSS, with Spearman’s correlation coefficient of 0.353 ( *p* < 0.001) and 0.457 ( *p* < 0.001), respectively. Median AVVQ score comparisons between groups with different degrees of problems by EQ-5D are shown in Table 2. AVVQ was able to discriminate patients with different levels of problems across all EQ-5D dimensions with significant differences in median AVVQ scores between groups in both pre-and postoperative periods except for postoperative self-care.

**Table 2.** Pre-and postoperative median AVVQ scores comparisons by percentage of patients reporting no problems for EQ-5D-3L.

EQ-5D dimension	AVVQ scores; median (IQR)					
	Preoperative			Postoperative		
	No problem	Some and severe problems	P value *	No problem	Some and severe problems	P value *
Mobility	14.4 (7.4, 22.3)	21.4 (14.5, 29.8)	0.001	5.0 (2, 8.2)	17.5 (8.3, 22.8)	<0.001
Selfcare	16.5 (9.7, 24.1)	26.1 (15.5, 40.0)	0.032	5.5 (2, 11)	13.9 (6.9, 20.7)	0.075
Usual activities	15.6 (9.1, 24.1)	19.7 (14.4, 27.6)	0.045	5.5 (2, 9.8)	19.2 (7, 25)	<0.001
Anxiety/depression	13.9 (7.3, 21.2)	19.7 (12.9, 29.4)	0.010	4.9 (2, 8.2)	13.8 (6.5, 20.7)	<0.001
Pain/discomfort	14.5 (7.4, 21.2)	22.3 (15.6, 27.6)	0.002	5.5 (2, 10)	14.8 (11.2, 20)	0.012

AVVQ, Aberdeen Varicose Vein Questionnaires; IQR, interquartile range.

* Wilcoxan rank-sum test.

AVVQ scores and VCSS improved significantly after operation in all severity classifications, see Table 3. Twenty-five patients (21%) reported having no problem for all EQ-5D dimensions preoperatively, whereas no patients had the lowest possible score for the AVVQ. For these 25 patients, there were changes in AVVQ scores with the pre-and postoperative median scores of 12.2 (IQR 5.5, 21.9) and 4.2 (IQR 0, 8.2) ( *p* = 0.002), respectively.

**Table 3.** Pre-and post-intervention AVVQ scores and VCSS according to CEAP clinical classification.

CEAP clinical classification	AVVQ scores; median (IQR)			VCSS; median (IQR)		
	Preoperative	Postoperative	P value *	Preoperative	Postoperative	P value *
C2 – C3	15.4 (8.3, 24.2)	4.2 (1.3, 8.4)	<0.001	5 (4, 7)	2 (1, 3)	<0.001
C4 – C6	18.9 (14.1, 25.5)	7.3 (4.6, 16.3)	<0.001	9 (7, 11)	4 (2, 6)	<0.001

AVVQ, Aberdeen Varicose Vein Questionnaires; IQR, interquartile range; VCSS, venous clinical severity scores.

The AVVQ is scored from 0-100 where 100 is the worst possible score. VCSS range from 0-30 where 30 is the worst possible score.

* Wilcoxan matched-pairs signed-rank test.

***Minimal clinically important differences (MCID)***

The MCIDs of the AVVQ varied from 2.18 to 17.16 across the four methods used ( Table 4). The lowest value was from accepted small ES MCID, which was 2.8, followed by 6.17, 6.21 and 17.16 from SD _observerd change_/2, SEM, and MDC _95_, respectively.

**Table 4.** Minimal clinically important differences (MCID) of the AVVQ scores from different distribution-based methods.

Methods		MCID
SD/2	SD _observed change_/2 = 12.35/2	6.17
SEM	SD _base_ × √((1)-r) = 10.9 × √(1-0.67)	6.21
MDC _95_	SEM ×1.96 × √2 = 6.21 ×1.96 × √2	17.16
Accepted small ES	SD _base_ × 0.2 =10.9 × 0.2	2.18

AVVQ, Aberdeen Varicose Veins Questionnaires; ES, effect size; MDC _95_, minimum detectable change with 95% confidence that this value is more than the measurement error; SD _base_, standard deviation of the baseline scores; SD _observed change_, standard deviation of the score changes following intervention; SEM, standard error of measurement.

## Discussion

Our study demonstrated acceptable internal consistency and moderate levels of reliability for the AVVQ-Thai. The AVVQ-Thai also had good construct and discriminative validity as demonstrated by the correlations with VCSS scores and significant differences of AVVQ scores between groups with different levels of health problems as assessed by EQ-5D. In addition, the AVVQ also had evidence of responsiveness to change following venous intervention.

AVVQ has been translated and validated in many languages, including Dutch, ^11^ Hungarian, ^25^ Spanish, ^26^ and Portuguese. ^27^ To our knowledge, the Thai-translated AVVQ (AVVQ-Thai) is the first translation in Asian countries. The Thai version also had good internal consistency comparable to other languages. ^10^^,^^25^^,^^27^ However, it had moderate test-retest reliability, which is less than the Portuguese AVVQ-Brazil, which had good test-retest reliability.

The AVVQ-Brazil study excluded patients more than 60 years old and had cognitive disorders (Mini-mental state examination), ^27^ while our study did not. About 43% of our patients were older than 60 years. Some older patients could not read the questionnaires independently due to eye problems and need for assistance. This problem might also have affected the results of retest AVVQ that demonstrated moderate test-retest reliability, which patients completed at home. Moreover, the different setting compared to test completion in the clinic, might also have led to slightly poorer results. Further studies, with the exclusion of older patients, can help delineate this problem.

Most previous studies compared AVVQ with SF-36 and demonstrated good discriminative validity and responsiveness of the AVVQ. ^2^^,^^9^^,^^11^^,^^25^^,^^27^ Our study used EQ-5D because of its lower respondent burden and it has been validated in Thai. ^28^ AVVQ-Thai also had good discriminative validity when using EQ-5D as a comparator too ( Table 2), except in the postoperative self-care domain. The proportion of patients reporting no problem in the self-care domain was not significantly different after venous intervention ( Figure 2). These findings suggest that self-care might not be affected by CVD.

Twenty-five patients (21%) had the highest possible EQ-5D score pre-intervention in the current study, which correspond with previous results study with CVD patients in Thailand. ^7^ Among these 25 patients, 10 (40%), 8 (32%), 5 (20%), and 2 (8%) were in CEAP C2, C3, C4, and C5, respectively, but the AVVQ can detect a change in these patients which shows the importance of applying AVVQ or other venous-specific QoL measurements alongside widely used generic PROMs such as the EQ-5D.

MCID is important for aiding the interpretation of score changes of PROMs including AVVQ and shows whether the difference is clinically meaningful and not just statistically significant. The estimates varied depending on the methods used but most studies recommend that SEM-derived MCID is acceptable as the most conservative approach, ^22^^,^^23^ which was 6.21. This estimate was very similar to 0.5*SD _observed change_-derived approach, ^24^ which was 6.17. ES-derived MCID, which was 2.18 in our study, likely to be an underestimate, while MDC _95_, which was 17.16, might be too high. Hence, in applications of the AVVQ in Thai patients, we recommend the MCID of 6.21 as a benchmark for clinical significance. However, this recommendation is subjected to further testing.

Our study had some limitations. The retest-AVVQ response rate of 79% while comparable to that from earlier test-retest studies, ^2^ was lower than that for clinic-based pre-and post-intervention completion. The number of included participants was less than the number from sample size calculation. We derived the MCID from the distribution-based approach. Other methods include anchor-based and sensitivity-specificity-based derivations. Results from the distribution-based approach were based on statistical variance measures, which meet statistical concepts, but might not reflect actual clinical significance differences. The other two methods compared the MCID with the clinical response measured by global transition questions (GTQ) to differentiate patients who are better or not, which might reflect better clinical significance. However, there is still no standard criteria for the GTQ, which weakens the validity of these methods.

## Conclusion

Thai-translated AVVQ had evidence for internal consistency, test-retest reliability, construct validity and responsiveness to changes in health following venous intervention. The AVVQ is recommended for application in Thailand for assessing the health and outcomes of patients with CVD.

**Preregistered data analysis**: The study was not preregistered.

### Ethics and consent

Ethic committees (The Human Research Ethics Committee of Thammasat University Medicine) with approval number MTU-EC-SU-1-130/61 (approved date July 26, 2018) for Thammasat University Hospital; The Research Ethics Committee 4 of Faculty of Medicine, Chiang Mai University with approval number SUR-2562-06329 (approved date June 28, 2019) for Maharaj Nakorn Chaing Mai Hospital; The Office of The Committee for Research, Faculty of Medicine Ramathibodi Hospital Mahidol University with approval number MURA2019/173 (approved date March 13, 2019) for Ramathibodi Hospital; The Research Ethic Committee of Chonburi Hospital with approval number 26/62/S/h3 (approved date March 20, 2019) for Chonburi Hospital.

All participants signed informed consent before entering the study.

## Data Availability

Zenodo.org: Reliability, validity, and minimal clinically important differences for the Thai-version of the Aberdeen Varicose Vein Questionnaire (AVVQ-Thai). https://doi.org/10.5281/zenodo.10990113. ^16^ This project contains the following underlying data:
• Checklist for Reliability, validity, and minimal clinically important differences for the Thai-version of the Aberdeen Varicose Vein Questionnaire (AVVQ-Thai) in patients with chronic venous disease, https://doi.org/10.5281/zenodo.10990113, file name: COSMIN checklist_AVVQ.docx. • The dataset for Reliability, validity, and minimal clinically important differences for the Thai-version of the Aberdeen Varicose Vein Questionnaire (AVVQ-Thai) in patients with chronic venous disease, https://doi.org/10.5281/zenodo.10990113, file name: AVVQ Thai raw data.dta • Reversed English-translated AVVQ for reversed English-translated AVVQ Reliability, validity, and minimal clinically important differences for the Thai-version of the Aberdeen Varicose Vein Questionnaire (AVVQ-Thai) in patients with chronic venous disease, https://doi.org/10.5281/zenodo.10990113, file name: Reverse-translated version.docx. • Final translated Thai AVVQ for Reliability, validity, and minimal clinically important differences for the Thai-version of the Aberdeen Varicose Vein Questionnaire (AVVQ-Thai) in patients with chronic venous disease, https://doi.org/10.5281/zenodo.10990113, file name: appendix 1_ThaiAVVQ.docx Data are available under the terms of the Creative Commons Zero v1.0 Universal (CC0).
