# Supplementary material for: Reliability, validity, and minimal clinically important differences for the Thai-version of the Aberdeen Varicose Vein Questionnaire (AVVQ-Thai) in patients with chronic venous disease
Source: F1000Res. 2024 Oct 11;13:486. Originally published 2024 May 17. [Version 2] doi: 10.12688/f1000research.147716.2 (PMC11803574; doi:10.12688/f1000research.147716.2)
Supplement: Peer review history

# Reviewer report: Reviewer response for version 2

**DOI:** 10.5256/f1000research.173215.r355673

**Published:** 2025-01-31

**Authors:** Tristan Lane

**Affiliations:** 1 Imperial College London & Cambridge University Hospitals, London, UK

Thank you for completing a difficult study to extend the options and standardisation of PROMs in varicose veins.

 The study has been completed well.

 The results and outcomes are presented well.

 Some comments:

 -Why did the study under-recruit?

 -Why was Eq-5D-3l not -5l used?  This would have reduced the ceiling effects seen.

 -The MCID is unfortunately hamstrung by the lack of data available - I appreciate this was to develop and validate the tool, but a larger cohort to repeat the validation and obtain the MCID would be beneficial.

 -What this completed on paper or on tablet?

 -Overall this is an excellent study well done.

Is the work clearly and accurately presented and does it cite the current literature?

Yes

If applicable, is the statistical analysis and its interpretation appropriate?

Yes

Are all the source data underlying the results available to ensure full reproducibility?

Yes

Is the study design appropriate and is the work technically sound?

Yes

Are the conclusions drawn adequately supported by the results?

Yes

Are sufficient details of methods and analysis provided to allow replication by others?

Yes

Reviewer Expertise:

vascular surgery, venous disease, varicose veins

I confirm that I have read this submission and believe that I have an appropriate level of expertise to confirm that it is of an acceptable scientific standard, however I have significant reservations, as outlined above.

# Reviewer report: Reviewer response for version 2

**DOI:** 10.5256/f1000research.173215.r343571

**Published:** 2025-01-08

**Authors:** Benilde Cosmi

**Affiliations:** 1 Angiology and Blood Coagulation Unit, Department of Medical and Surgical Sciences, University of Bologna; IRCCS Azienda Ospedaliera Universitaria di Bologna, University of Bologna, Bologna, Italy

Siribumrungwong et al. Conducted a multicenter prospective observational cohort study from January 2019 to Dec 2021 to assess the reliability and validity of the Thai-translated version of the Aberdeen Varicose Vein Questionnaire (AVVQ)

 Methods

 participants: The authors should specify who evaluated the CEAP class of venous insufficiency, how the pathological reflux of the great or small saphenous vein was assessed and which were the indication for venous interventions.

 Results:

 The calculated sample size was 145 patients, however only 119 were recruited. The authors should explain this discrepancy.

 Results: The majority of patients had CEAP class 2 and 3 (59%- the lowest severity ) and thus the indication for intervention may be questionable as in these cases conservative medical management could be indicated. The authors should comment on this issue, as it would be expected that patients with CEAP class 4-6 would have more benefit from surgical intervention and thus pre and post-surgical  AVVD questionnaire could show a relevant improvement.

Is the work clearly and accurately presented and does it cite the current literature?

Yes

If applicable, is the statistical analysis and its interpretation appropriate?

I cannot comment. A qualified statistician is required.

Are all the source data underlying the results available to ensure full reproducibility?

Yes

Is the study design appropriate and is the work technically sound?

Partly

Are the conclusions drawn adequately supported by the results?

Partly

Are sufficient details of methods and analysis provided to allow replication by others?

Partly

Reviewer Expertise:

angiology, venous thromboembolism,

I confirm that I have read this submission and believe that I have an appropriate level of expertise to confirm that it is of an acceptable scientific standard, however I have significant reservations, as outlined above.

# Reviewer report: Reviewer response for version 2

**DOI:** 10.5256/f1000research.173215.r330857

**Published:** 2024-10-12

**Authors:** Nawaphan Taengsakul

**Affiliations:** 1 Chulabhorn Hospital, Bangkok, Thailand

Excellent research design that can be used as the Thai population's standard AVVQ question. Excellent writing and evaluation, together with the right statistical analysis.

Is the work clearly and accurately presented and does it cite the current literature?

Yes

If applicable, is the statistical analysis and its interpretation appropriate?

Yes

Are all the source data underlying the results available to ensure full reproducibility?

Yes

Is the study design appropriate and is the work technically sound?

Yes

Are the conclusions drawn adequately supported by the results?

Yes

Are sufficient details of methods and analysis provided to allow replication by others?

Yes

Reviewer Expertise:

Vascular surgery, venous surgery, general surgery

I confirm that I have read this submission and believe that I have an appropriate level of expertise to confirm that it is of an acceptable scientific standard.

# Reviewer report: Reviewer response for version 1

**DOI:** 10.5256/f1000research.161943.r313678

**Published:** 2024-09-03

**Authors:** Masumeh Hessam

**Affiliations:** 1 Ahvaz Jundishapur University of Medical Sciences, Ahvaz, Iran

1: Please reorganize the presentation of method and results starting by internal consistency, reliability, continuing by construct validity in whole manuscript.

 2: The introduction cannot explain the gap in the current researches.

 3: Since the purpose of this study is to investigate the psychometric properties of the instrument, it is recommended to give a brief description of these properties (validity and reliability) in introduction section.

 4: Discussion lacks some clarity. I think you should discuss your results and not explained them. So it can be re-write for explaining the results, not just presenting them.

 5: Authors should be mentioned suggestions for future studies (such as investigating responsiveness and the ability of the questionnaire to detect clinical changes) in the discussion section.

Is the work clearly and accurately presented and does it cite the current literature?

Partly

If applicable, is the statistical analysis and its interpretation appropriate?

I cannot comment. A qualified statistician is required.

Are all the source data underlying the results available to ensure full reproducibility?

No source data required

Is the study design appropriate and is the work technically sound?

Yes

Are the conclusions drawn adequately supported by the results?

Yes

Are sufficient details of methods and analysis provided to allow replication by others?

Yes

Reviewer Expertise:

psychometric propertis of outcome measures in rehabilitation.

I confirm that I have read this submission and believe that I have an appropriate level of expertise to confirm that it is of an acceptable scientific standard, however I have significant reservations, as outlined above.

# Response

**Published:** 2024-10-08

**Authors:** Boonying Siribumrungwong

**Affiliations:** Surgery, Thammasat University, Pathum Thani, Thailand

1: Please reorganize the presentation of method and results starting by internal consistency, reliability, continuing by construct validity in whole manuscript.

 Response: We have organized the method and results section in order as the suggestion.

 2: The introduction cannot explain the gap in the current researches.

 Response: We have included a sentence in the introduction addressing the limited data available on the minimal clinically important difference for AVVQ. This data remains insufficient to fully explain the knowledge gap that this study aims to address.

 3: Since the purpose of this study is to investigate the psychometric properties of the instrument, it is recommended to give a brief description of these properties (validity and reliability) in introduction section.

 Response: As you suggested, we have added brief descriptions of validity, reliability, and responsiveness in the introduction section according to the COSMIN guidelines.

 4: Discussion lacks some clarity. I think you should discuss your results and not explained them. So it can be re-write for explaining the results, not just presenting them.

 Response: Thank you for the comment. We tried our best to discuss the test-retest reliability more in the discussion part.

 5: Authors should be mentioned suggestions for future studies (such as investigating responsiveness and the ability of the questionnaire to detect clinical changes) in the discussion section.

 Response: We have added some suggestions for future studies in the discussion section.

# Reviewer report: Reviewer response for version 1

**DOI:** 10.5256/f1000research.161943.r289501

**Published:** 2024-06-20

**Authors:** Nawaphan Taengsakul

**Affiliations:** 1 Chulabhorn Hospital, Bangkok, Thailand

Siribumrungwong B and co-authors developed the Aberdeen Varicose vein Questionnaire (AVVQ) in Thai language that would be the first validated questionnaire for evaluating chronic venous disease in Thailand. The AVVQ is the patient-reported outcome measure that is used widely and well accepted in reliability and validity as recommended in many guideline such as ESVS guideline for reporting. Therefore, It is important to translate for usable in their language. Moreover, The AVVQ-Thai had the satisfactory result that can be useful and reproducible for further study in Thailand.

 I think this paper is well conducted, and the manuscript is certainly well written. The strengths of this research was the translation process and the statistical analysis. But I see some details that should be clarified.

 First of all, it should be added about pilot test after the forward, backward translation and expert review that could be necessary for represent about usability for patients before distribution.

 Seconds, the recent guideline recommended change to r-VCSS that prove that more reliability. The authors should clarify why to used VCSS, not r-VCSS to compare.

 The third problem, this study did not exclude the elderly that might effect to ability for answering questionnaire especially when compared with AVVQ-Brazil study. In part of discussion, may be added that the further study with excluded the patient with impaired sensation should be considered.

 Lastly, the successful operation should be defined such as the evidence of target vein occlusion (<2 cm from junction and >90% occluded vein) by ultrasound

Is the work clearly and accurately presented and does it cite the current literature?

Yes

If applicable, is the statistical analysis and its interpretation appropriate?

Yes

Are all the source data underlying the results available to ensure full reproducibility?

Yes

Is the study design appropriate and is the work technically sound?

Yes

Are the conclusions drawn adequately supported by the results?

Yes

Are sufficient details of methods and analysis provided to allow replication by others?

Yes

Reviewer Expertise:

Vascular surgery, venous surgery, general surgery

I confirm that I have read this submission and believe that I have an appropriate level of expertise to confirm that it is of an acceptable scientific standard.

# Response

**Published:** 2024-10-07

**Authors:** Boonying Siribumrungwong

**Affiliations:** Surgery, Thammasat University, Pathum Thani, Thailand

**Reviewer comment: **1. First of all, it should be added about pilot test after the forward, backward translation and expert review that could be necessary for represent about usability for patients before distribution.               

**Author response: **Thank you for your comments. The translation was independently done by two professionals translators, consensus by the research team, and reversed to English by a native English speaker. We have added the reversed English translation for review in the data availability.

**Reviewer comment: **2. Seconds, the recent guideline recommended change to r-VCSS that prove that more reliability. The authors should clarify why to used VCSS, not r-VCSS to compare.

**Author response: **Thank you for your comments. We must apologize for the missed clarification. We had already used the revised version of the VCSS in the study. We have rewritten it in the manuscript to be more clarified and added a reference for it.

**Reviewer comment: **3. The third problem, this study did not exclude the elderly that might effect to ability for answering questionnaire especially when compared with AVVQ-Brazil study. In part of discussion, may be added that the further study with excluded the patient with impaired sensation should be considered.

**Author response: **We have revised the study based on your comments.

**Reviewer comment: **4. Lastly, the successful operation should be defined such as the evidence of target vein occlusion (<2 cm from junction and >90% occluded vein) by ultrasound

**Author response: **We have added the definition of successful operation in the manuscript.
